# Abdominal endograft collapse due to acute type A aortic dissection

**DOI:** 10.1007/s10047-025-01533-8

**Published:** 2025-11-12

**Authors:** Satoki Nakamura, Tomoaki Kudo, Junki Yokota, Noriko Kodani, Masatoshi Hata, Toru Kuratani

**Affiliations:** Department of Cardiovascular Surgery, Osaka International Medical and Science Center, 10-31, Karasugatsuji, Osaka-shi Tennoji-ku, Osaka, Osaka 543-8922 Japan

**Keywords:** Endovascular aortic repair, Aortic dissection, Malperfusion

## Abstract

Endovascular repair (EVAR) for abdominal aortic aneurysm and iliac aneurysm is a safe and valid treatment. However, it is associated with some different complications that may require reinterventions. Among these complications, abdominal endograft proximal collapse is an infrequent event. We report a case of acute type A aortic dissection and abdominal endograft proximal collapse in a patient previously treated with EVAR.

## Introduction

Acute type A aortic dissection (TAAD) is a life-threatening condition with high mortality that requires emergency treatment. Despite the increasing utilization of endovascular aneurysm repair (EVAR), the occurrence of new-onset TAAD after EVAR is extremely rare [1]. Its presence complicates the management of aortic dissection and can lead to severe outcomes, including the collapse of the pre-existing endograft. We report a case of acute type A aortic dissection complicated by proximal collapse of an abdominal endograft in a patient with a history of EVAR.

## Case report

All procedures in this study were conducted according to the principles of the Declaration of Helsinki and were approved by the Medical Ethics Committee of Osaka International Medical and Science Center (No. 1536). We obtained informed consent from this patient in writing before we performed the procedures.

**Fig. 1:**
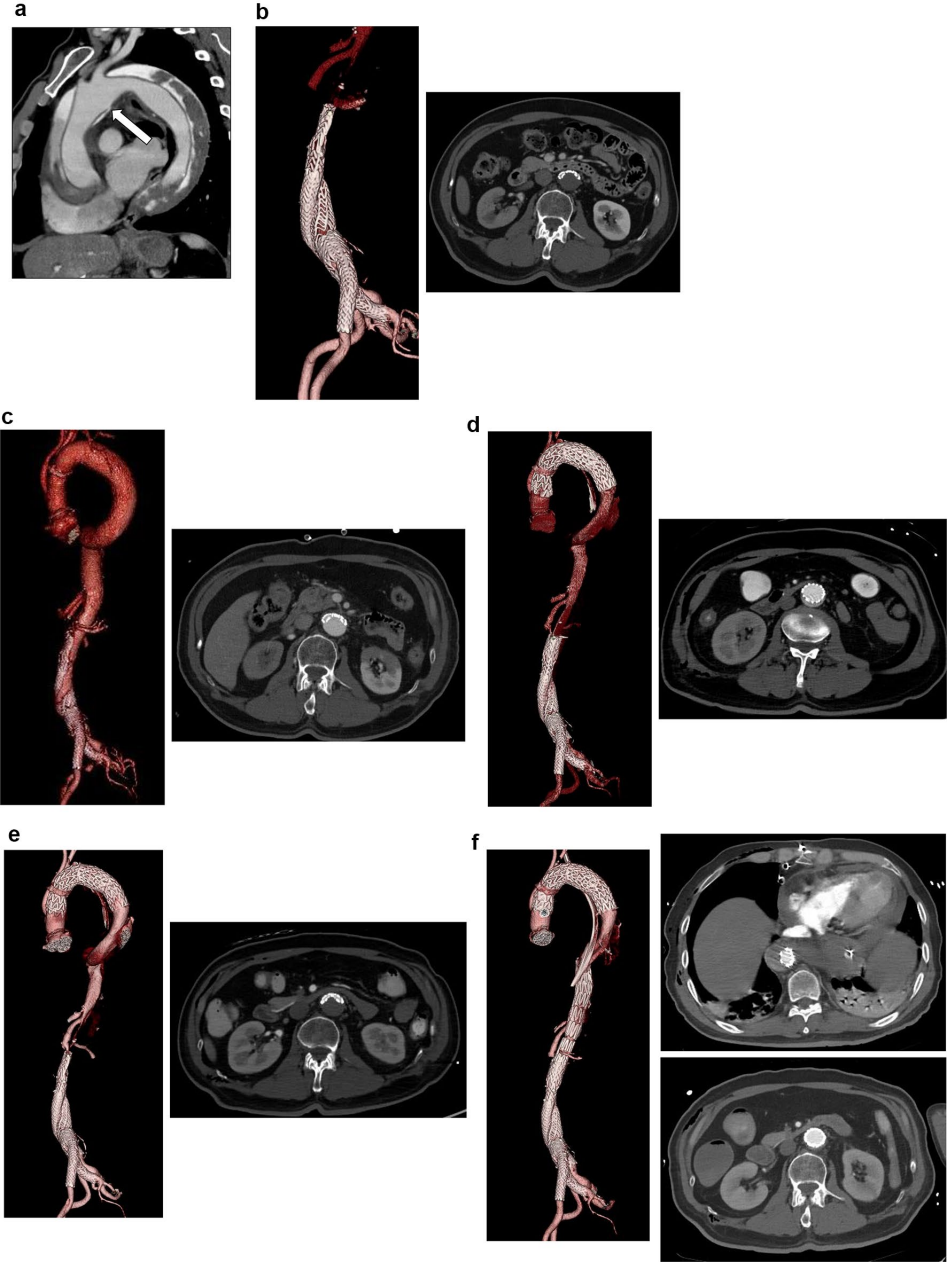
Computed tomography angiography (CTA). **A** Preoperative CTA; primary entry at arch (arrow). **B** Preoperative CTA; collapse of EVAR endograft. **C** Postoperative CTA after graft replacement; collapse of EVAR endograft. **D** Follow-up CTA after TEVAR; expansion of EVAR endograft. **E** Follow-up CTA; re-collapse of EVAR endograft. **F** CTA after placement of a PETTICOAT device. EVAR; endovascular aortic repair, TEVAR; thoracic endovascular aortic repair

A 75-year-old man with a history of hypertension was presented to the emergency room with back pain. His medical history included an EVAR for a left common iliac artery (CIA) aneurysm using a GORE EXCLUDER device (W. L. Gore & Associates, Flagstaff, AZ, USA) 5 years prior without any evidence of endoleak or significant problems. A computed tomography angiography (CTA) revealed type A aortic dissection (TAAD) extending from the sinotubular junction to the right external iliac artery, which had the main entry at an aortic arch between brachiocephalic artery (BCA) and left common carotid artery (LCCA) and no significant re-entry tears (Fig. 1A). The main body of the EVAR endograft, the descending and thoracoabdominal aorta, and the abdominal aorta were compressed by the false lumen of the dissection, resulting in severe lower body malperfusion syndrome (Fig. 1B). Given the emergent and life-threatening nature of the TAAD with malperfusion, we performed an emergent partial arch replacement with BCA and LCCA reconstructions to resect the main entry. We thought that by resecting the main entry, the false lumen blood flow would disappear, thereby leading to the resolution of external compression and maintaining the patency of the EVAR endograft.

**Fig. 2:**
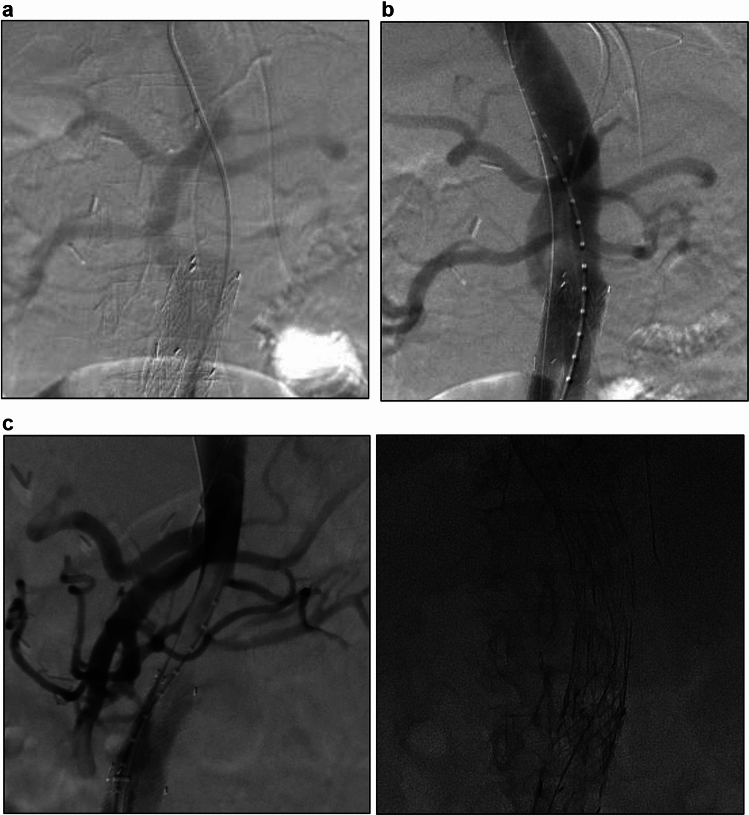
Intraoperative angiography. **A** Defect of contrast medium before TEVAR. **B** Expansion of the collapsed endograft after TEVAR. **C** Defect of contrast medium before placement of a PETTICOAT device. **D** Expansion of the collapsed endograft after placement of a PETTICOAT device. TEVAR; thoracic endovascular aortic repair

On the following day, blood pressure in both legs abruptly decreased. Subsequent CTA showed that the main body of the EVAR endograft, the descending and abdominal aorta, and both legs were persistently compressed at the same level as before surgery by the remaining false lumen blood flow from the anastomosis of the artificial blood vessel (Fig. 1C). Stent-induced new entry was not observed. Thoracic endovascular aortic repair (TEVAR) was performed to close the entry tear at the anastomosis site, followed by ballooning to expand the collapsed main body of EVAR endograft. An intraoperative angiography confirmed successful expansion of the collapsed endograft (Fig. 2A, B). Seven days later, a follow-up CTA showed re-expansion of the true lumen, the descending and thoracoabdominal aorta, and the abdominal endograft (Fig. 1D). On the next day after this CTA, blood pressure in both legs suddenly decreased precipitously again. The CTA revealed that the EVAR endograft, the descending and thoracoabdominal aorta, and both legs were compressed at the same level as before surgery (Fig. 1E). To restore true lumen blood flow, we deployed a PETTICOAT (Cook Medical, Bloomington, IN, USA) from the descending aorta to the main body of the endograft (Fig. 2C, D). Postoperative CTA after the procedure demonstrated re-expansion of the infrarenal endograft and improved blood flow to the lower extremities, with minimal residual flow to the false lumen. Despite these interventions, the patient died 3 days later from massive hematemesis and hemorrhagic shock secondary to aortic rupture and aortoenteric fistula. Due to a lack of family consent, this post-mortem study was not performed. We inferred the cause of death based on the clinical course and CT findings (Fig. 1F).

## Discussion

EVAR has become a widely utilized as a minimally invasive treatment option for abdominal aortic aneurysms [2]. Aortic dissection is accompanied by poor perfusion due to excessive pressure in the false lumen and lack of sufficient outflow, which can lead to severe outcomes such as false lumen expansion, rupture, and endograft collapse. However, EVAR endograft collapse due to aortic dissection is a rare complication. Its incidence is unknown, and its causes are not fully understood. The mortality rate for this specific complication (EVAR endograft collapse in the context of aortic dissection) has been reported to be as high as 42% [3]. The causes of endograft collapse include inappropriate sizing, deterioration, mechanical stress, and arterial remodeling. It has been reported that re-expansion with additional stent graft placement is effective [4, 5].

In this case, treatment for the type A dissection was required simultaneously with treatment for lower limb malperfusion resulting from EVAR endograft collapse. We thought that the main entry could be resected by performing artificial vascular replacement, the false lumen blood flow would disappear, and the EVAR endograft would remain patent. However, despite the primary entry resection, the false lumen blood flow did not disappear due to the formation of a new entry at the distal anastomosis of the artificial vascular graft, resulting in continued lower limb malperfusion. Although our strategy is primary entry resection, the use of a frozen elephant trunk (FET) might have provided a more secure solution in this case. Subsequently, the lower limb malperfusion was temporarily improved by expanding the TEVAR and EVAR endograft, but the EVAR endograft collapsed again, resulting in lower limb malperfusion. Reported treatment strategies for collapsed EVAR endograft include: (1) graft replacement, (2) TEVAR with primary entry closure, (3) extra-anatomical bypass, and (4) stenting for the endograft main body [5, 6]. We considered replacing the EVAR endograft with an artificial vascular graft as a treatment at this time. Still, we determined that this would be too invasive, so we decided to expand the EVAR endograft by ballooning.

Although this procedure initially showed some improvement, the EVAR graft subsequently collapsed again, and we reduced blood flow to the lower extremities rapidly. Because we attributed this recollapse and reduced blood flow to structural deterioration of the EVAR endograft, we decided to place a PETTICOAT stent to reduce the deformation of the EVAR graft and maintain true lumen patency. This intervention initially showed radiographic and clinical improvement in true lumen expansion and blood flow.

However, the patient ultimately died of aortic rupture. From this tragic outcome, we concluded that despite the small amount of false lumen blood flow from the minor entry remaining in the descending aorta, the false lumen became a closed compartment, causing a critical increase in false lumen pressure, which ultimately led to aortic rupture. From this perspective, the EVAR endograft collapse after EVAR endograft expansion might have been not only due to structural deterioration of the EVAR endograft, but also due to an increase in false lumen pressure caused by the minor entry remaining in the descending aorta.

In cases where the EVAR graft is deformed, such as this case, simply expanding the EVAR graft with a balloon may result in re-deformation. It is essential to identify the cause of the deformation and address it effectively. This highlights the importance of precisely identifying and addressing the source of false lumen pressurization when managing endograft collapse in the context of aortic dissection.

## Conclusion

In some cases, aortic dissection occurring after EVAR can result in endograft collapse and subsequent aortic rupture, leading to a fatal outcome. While primary entry closure using endovascular only approaches can be effective in some cases, a more comprehensive treatment strategy, including open surgical repair (total arch replacement with or without FET), should be considered in patients with high false lumen pressure.
